# Usefulness of the l‐type Wako *Helicobacter pylori* antibody J test

**DOI:** 10.1002/jgh3.12553

**Published:** 2021-05-07

**Authors:** Yoshitaka Tokai, Junko Fujisaki, Naoki Ishizuka, Hiroki Osumi, Ken Namikawa, Shoichi Yoshimizu, Yusuke Horiuchi, Akiyoshi Ishiyama, Toshiyuki Yoshio, Toshiaki Hirasawa, Kazumasa Miki, Tomohiro Tsuchida

**Affiliations:** ^1^ Department of Gastroenterology The Cancer Institute Hospital of Japanese Foundation for Cancer Research Tokyo Japan; ^2^ Department of Clinical Trial planning and Management The Cancer Institute Hospital of Japanese Foundation for Cancer Research Tokyo Japan

**Keywords:** ELISA, *Helicobacter pylori*, latex agglutination tests

## Abstract

**Background and Aim:**

*Helicobacter pylori* antibody levels in the blood are currently measured using an ELISA. In April 2016, FUJIFILM Wako Pure Chemical Corporation launched the “l‐type Wako *Helicobacter pylori* antibody J" test, which is based on the latex agglutination turbidimetric immunoassay. In this study, we investigated the usefulness of the Wako test.

**Methods:**

We measured *H. pylori* antibody levels using both the ELISA and Wako test in 180 patients who underwent upper gastrointestinal endoscopy at our hospital between September 2017 and February 2019. Ninety patients were infected with *H. pylori*. We calculated the diagnostic accuracy, sensitivity, and specificity of each test and the concordance rate between the ELISA and Wako test. If lower limits of 90% confidence intervals (CIs) for each diagnostic validity exceeded the 85% threshold, the usefulness of the diagnostic test was confirmed.

**Results:**

The diagnostic accuracy, sensitivity, and specificity were 94.4% (90% CI, 90.8–97.0%), 94.4% (90% CI, 88.7–97.8%), and 94.4% (90% CI, 88.7–97.8%), respectively, when the Wako test was used, and 94.4% (90% CI, 90.8–97.0%), 88.9% (90% CI, 81.9–93.8%), and 100% (90% CI, 96.0–100%), respectively, when the ELISA was used. The concordance rate between the two tests was high (*κ* = 0.8444).

**Conclusions:**

We confirmed the usefulness of the Wako test, especially when screening for *H. pylori* infection, due to its high sensitivity.

## Graphical abstract

“l‐type Wako *Helicobacter pylori* antibody J” test is based on the latex agglutination turbidimetric immunoassay. Many samples can be analyzed simultaneously, rapidly, and more economically using this kit. l‐HP J test showed a high diagnosis performance, and is not inferior to ELISA. The l‐HP J test is useful, especially when screening for *Helicobacter pylori* infection, due to its high sensitivity.

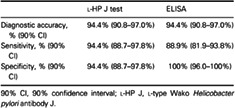

## Introduction

*Helicobacter pylori* (*H. pylori*), a gram‐negative bacillus, was discovered in 1983 by Warren and Marshall and is known to persistently infect the human gastric mucosa and induce histological gastritis accompanied by neutrophilic infiltration.^1^ It has been demonstrated that *H. pylori* infection is associated with the development of gastric ulcer/duodenal ulcer and gastric cancer.^2^, ^3^ People in Japan were notified that eradication therapy for *H. pylori*‐infected gastritis would be covered by health insurance starting February 2013; thus, diagnosing *H. pylori* infection became more prevalent.

Diagnostic methods for *H. pylori* infection with endoscopy include culture test, microscopic test, and rapid urease test, and the tests without endoscopy include urea breath test (UBT), serous *H. pylori* antibody test, and feces *H. pylori* antigen test.^3^, ^4^

The serous *H. pylori* antibody test is convenient and is widely used to diagnose *H. pylori* infection in the current clinical practice. The prevailing methods for this antibody test are the ELISA (Eiken Chemical, Tokyo, Japan) and the chemiluminescent method, both of which have limitations: they take a long time for analysis and require special measuring devices.

In April 2016, FUJIFILM Wako Pure Chemical Corporation launched the “l‐type Wako *H. pylori* antibody J" (l‐HP J) test (FUJIFILM Wako Pure Chemical Corporation, Osaka, Japan), which is based on the latex agglutination turbidimetric immunoassay.

**Figure 1:**
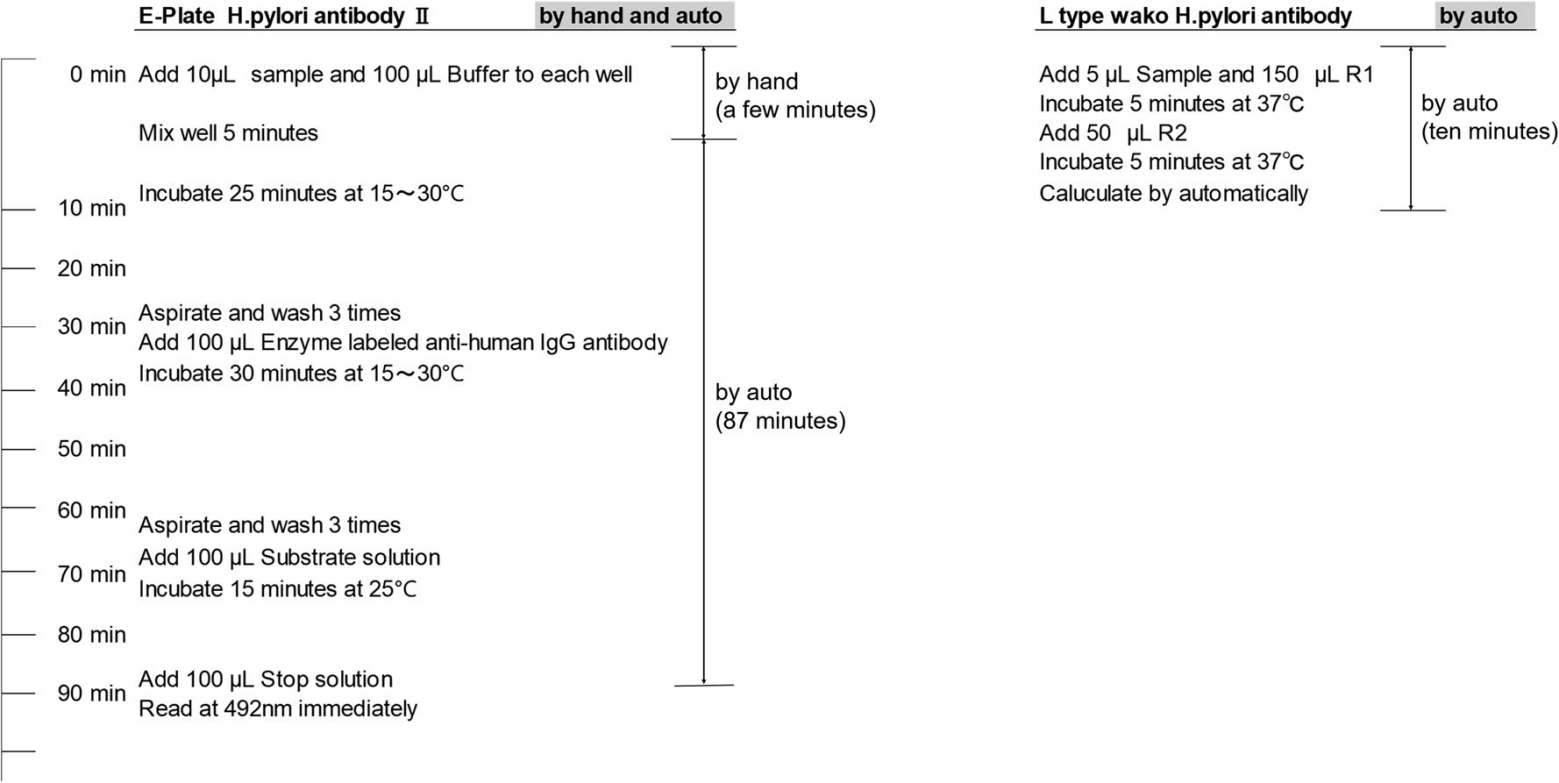
Assay procedure of the E‐Plate *Helicobacter pylori* antibody II (ELISA) and l‐HP･J tests for one sample. ELISA takes 90 min “by hand and auto” per plate (1–96 samples). In contrast, a biochemical autoanalyzer (L type Wako *H. pylori* antibody) requires 10 min for each sample. l‐HP J, l‐type Wako *Helicobacter pylori* antibody J.

Since this kit can be used with a general‐purpose automatic analyzer, it is less complicated than ELISA. Many samples can be analyzed simultaneously, rapidly, and more economically using this kit (Fig. 1). Using this kit, the presence of *H. pylori* infection can be diagnosed and be prescribed drugs to eradicate *H.pylori* on the day of the blood test. Consequently, this kit might contribute to reduced medical costs. However, its accuracy has not been confirmed. In this study, we evaluated the diagnostic validity of the l‐HP J test, which has not been demonstrated before, by calculating its diagnostic accuracy, sensitivity, specificity, area under the curve (AUC), and concordance rate with ELISA.

## Methods

### Subjects

This observational, validation study was conducted in clinical settings, and included consecutive patients who underwent gastrointestinal endoscopy between September 2017 and February 2019. The following patients were excluded: those who had received eradication therapy for *H. pylori* or had undergone gastrectomy; those who had taken proton pump inhibitors within 2 weeks before undergoing the UBT; those who had taken antibiotics within 4 weeks before the endoscopy; those who had undergone head and neck surgery and were unable to undergo the UBT; those in whom advanced gastric cancer was detected endoscopically; and those who were suspected of having type A gastritis endoscopically (cases with severe atrophic gastritis of the corpus and mild or no atrophic gastritis of the antrum). Our medical staff interviewed the patients to determine whether they had received eradication therapy for *H. pylori* or had undergone gastrectomy in the past. Major reasons for undergoing gastrointestinal endoscopy included screening, testing positive for *H. pylori* antibodies, having abdominal symptoms such as upper abdominal pain, and workup for early gastric cancer.

### Serum H. pylori kits and assay procedure

We measured *H. pylori* antibodies in‐house, in specimens from subjects who provided consent, using (i) the l‐HP J test and (ii) the E‐plate “Eiken” *H. pylori* antibody test (ELISA). Blood sampling was done on the day endoscopy was performed. Cutoff values used for these antibody tests were as follows: (i) l‐HP J test, 4 U/mL or more was considered positive for *H. pylori* (This is a validation study of l‐HP J test with an already confirmed cut‐off value of 4 U/mL. Therefore, we used this accepted cut‐off value [4 U/mL] in this study), and (ii) ELISA, 10 U/mL or more was considered positive for *H. pylori*. Measurement using each kit was performed in‐house. In this study, we used a full auto microplate chemiluminescence analyzer (Hitachi Chemical Diagnostics Systems Co., Ltd. Tokyo, Japan) for ELISA, and Hitachi Labospect 008 automatic biochemistry analyzer (Hitachi High‐Technologies) for the l‐HP J test. There are two analyzing processes for ELISA (Fig. 1); “by hand” and “by auto.” First, dispensing a specimen and buffering to the container “by hand” is needed, and lasts few min for each sample (it depends on the engineer's skills). After that, ELISA requires bound (B)/free (F) antibodies to be separated by washing. This process is performed “by auto” and requires approximately 87 min to analyze 1–96 specimens. In contrast, the latex agglutination turbidimetric immunoassay does not require a similar separation; hence, one specimen can be analyzed in 10 min, and the results of 100 specimens can be obtained in 16 min. Reagents were provided by the FUJIFILM Wako Pure Chemical Corporation. Fresh plasma was used for the measurement of ELISA, and cryopreserved sera (−20°C) taken within 4 weeks or fresh plasma were thawed and used for the measurement of l‐HP J test.

### Endoscopic findings

Esophagogastroduodenoscopy (EGD) was performed with the Evis Lucera Elite system (Olympus Corporation, Tokyo, Japan) and a GIF‐H290Z scope (Olympus Corporation). The grading of gastric mucosal atrophy by endoscopic findings was based on the Kimura–Takemoto classification^5^: no atrophy (C‐0); closed‐type (C‐1 to C‐3), in which an endoscopic atrophic border is in the lesser curvature of the gastric body and does not extend beyond the cardia; and open type (O‐1 to O‐3), in which an endoscopic atrophic border extends beyond the cardia and progresses into the greater curvature.

**Figure 2:**
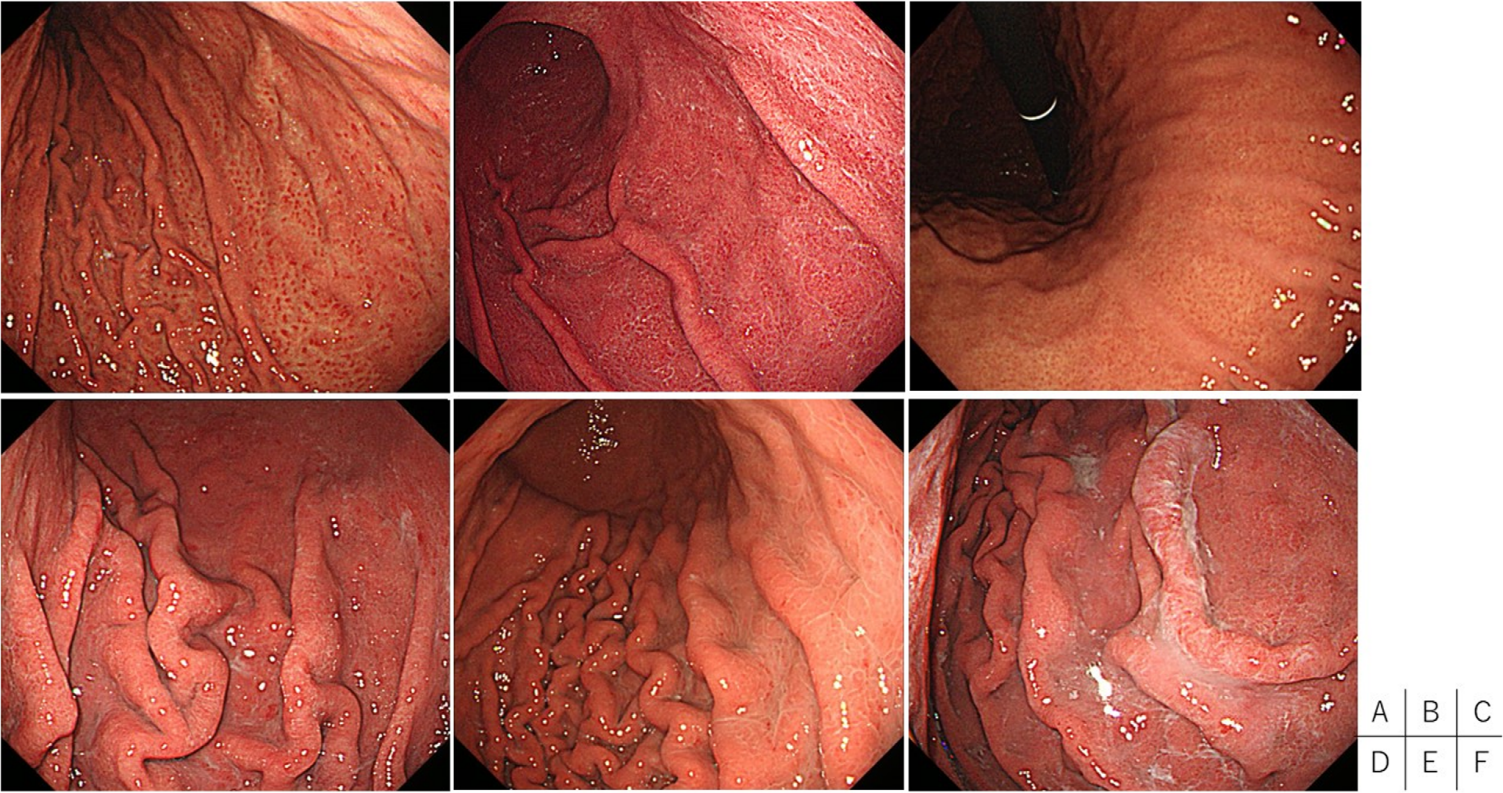
Endoscopic findings that suggest active gastritis. (a) Spotty redness: multiple tiny reddish spots in the fundic gland region. Spotty redness is usually observed in the upper region of the stomach. (b) Diffuse redness: uniform redness involving the entire mucosa of the fundic gland region. (c) Disappearance of the regular arrangement of collecting venules: starfish‐like red spots in a regular arrangement visible through the mucosal surface in the fundic gland region have disappeared due to *H. pylori*‐induced inflammation. (d) Enlarged/tortuous folds. (e) mucosal swelling. (f) sticky mucus.

Endoscopic findings, which suggest active gastritis, such as diffuse redness, mucosal swelling, sticky mucus, and enlarged fold, are reported to have high diagnostic odds ratio and are helpful in the diagnosis of current *H. pylori* infection.^6^, ^7^ Therefore, in determining *H. pylori* infection status, we used these findings (Fig. 2).

### Criteria for determining H. pylori infection status and methods to evaluate the kits

*H. pylori* infection was diagnosed by integrating the endoscopic findings and the UBT results as follows:

Subjects who had no gastric mucosal atrophy with no findings suggesting active gastritis on EGD as described above and whose UBT result was negative (2.5% or more is defined positive) (UBIT, Otsuka Pharmaceutical) were considered uninfected, while those who had findings of either gastric mucosal atrophy, or two or more active gastritis detected on EGD with positive UBT result were considered infected. Patients whose endoscopic findings and UBT results had a discrepancy, such as active gastritis detected endoscopically, indicating a current *H. pylori* infection, but had negative UBT results, and conversely, patients who were not infected according to their endoscopic findings but whose UBT results were positive, were excluded from the study.

Subjects whose endoscopic findings and UBT results concurred in terms of the presence/absence of *H. pylori* infection were diagnosed as being positive or negative for *H. pylori*, and the sensitivity, specificity, and diagnostic accuracy of the l‐HP J test were determined. ELISA was performed in a similar manner, and the concordance rate with the l‐HP J test was calculated. In cases misdiagnosed by l‐HP J test, the test results correlated with the clinical findings.

### Sample size calculation

According to a previous report from Japan, the sensitivity and specificity of l‐HP J test for *H. pylori* positive cases were 96.4% (currently infected; 56 cases) and 97.7% (uninfected; 86 cases), respectively. Therefore, we set an expected value as 95%, and threshold value as 85% for both, sensitivity and specificity. Based on the binomial test, a sample size of 85 cases was deemed to be required for both uninfected and currently infected cases, using the following criteria: one‐sided alpha of 5%; and power of 90%. Ultimately, a total of 180 patients (90 uninfected and 90 currently infected cases) were enrolled to make up for any dropouts.

### Statistical analyses

All continuous variables were expressed as the median and range. For diagnostic performance, diagnostic accuracy, sensitivity, and specificity are presented as percentages with 90% confidence interval (90% CI). To evaluate concordance between the test methods for positivity and negativity, the *κ* statistic was used. The McNemar's test, which evaluates marginal homogeneity between two test methods, was used to compare the l‐HP J test and ELISA. To assess the diagnostic accuracies of the l‐HP J test and ELISA, a receiver‐operating characteristic (ROC) curve was constructed by plotting sensitivity *versus* 1‐specificity values for both the l‐HP J test and ELISA and its AUC was computed. Subsequently, we compared the AUC values with 95% CIs obtained for the l‐HP J test and ELISA using *t*‐tests. A *P* value <0.05 was considered statistically significant. Statistical analysis was performed using Statistical Analysis System (SAS) software 9.4 (TS1M5, SAS Institute Inc., Cary, North Carolina).

### Ethics

This study was approved by the hospital's institutional review board, and written informed consent was obtained from all subjects.

## Results

### Breakdown of H. pylori infection status

Among 180 patients, 90 patients (50%) were uninfected and 90 patients (50%) had a *H. pylori* infection. Among the patient characteristics between uninfected and currently infected cases, the median age and percentage of male patients were 57 years (range; 37–76), and 65 years (range; 28–87), 45.6% and 57.8%, respectively. The uninfected group was younger. Among the currently infected subjects, the degree of gastric mucosal atrophy (the Kimura–Takemoto classification) was C‐1 to C‐3 in 51.1% (46/90) and O‐1 to O‐3 in 48.9% (44/90). Although the major comorbidity among the currently infected subjects was early gastric cancer, this was seen only in one patient among the uninfected subjects (Table 1).

**Table 1:** Characteristics of subjects with current infection and no infection with *Helicobacter pylori*

	Uninfected *n* = 90	Currently infected *n* = 90
Age, median (range)	57 (37–76)	65 (28–87)
Sex, *n* (%)		
Male	41 (45.6%)	52 (57.8%)
Female	49 (54.4%)	38 (42.2%)
Degree of atrophy, *n* (%) no atrophy	90 (100%)	0 (0%)
Closed‐type atrophy	0 (0%)	46 (51.1%)
Open‐ type atrophy	0 (0%)	44 (48.9%)
Titer of l‐HP J test (U/mL), median (range)	0.75 (0–13.6)	32.7 (2.7–109.8)
UBT (‰), median (range)	0.4 (0–1.6)	13.8 (2.7–135.3)
Comorbidity		
Early gastric cancer, *n* (%)	1 (1.1%)	28 (31.1%)
Superficial esophageal cancer, *n* (%)	1 (1.1%)	5 (5.6%)
Duodenal cancer, *n* (%)	1 (1.1%)	0 (0%)
Post‐ESD for early gastric cancer, *n* (%)	0 (0%)	2 (2.2%)
Post‐ESD for superficial esophageal cancer, *n* (%)	0 (0%)	1 (1.1%)
Gastric ulcer scar, *n* (%)	0 (0%)	1 (1.1%)

Data are presented as number (%).

l‐HP J, l‐type Wako *Helicobacter pylori* antibody J; UBT, urea breath test.

### Diagnostic validity of the l‐HP J test (diagnostic accuracy, sensitivity, and specificity)

The diagnostic accuracy, sensitivity, and specificity for current *H. pylori* infection were 94.4% (90% CI; 90.8–97.0%), 94.4% (90% CI; 88.7–97.8%), and 94.4% (90% CI; 88.7–97.8%), respectively, when the l‐HP J test was used, and 94.4% (90% CI; 90.8–97.0%), 88.9% (90% CI; 81.9–93.8%), and 100% (90% CI; 96.0–100%), respectively, when ELISA was used (Table 2). Although the lower limits of 90% CIs of the l‐HP J test exceeded the threshold value (85%) in terms of diagnostic accuracy, sensitivity, and specificity, the lower limits of 90% CIs for sensitivity of ELISA (81.9%) were lower than the threshold value (85%). Furthermore, the calculated AUC values were compared. AUC between the l‐HP J test (AUC = 0.994, 95% CI = 0.988–1) and ELISA (AUC = 0.988, 95% CI = 0.972–1) showed no statistical significance (*P* = 0.459).

**Table 2:** Diagnostic validity of the l‐type Wako *Helicobacter pylori* antibody J test and the ELISA

	l‐HP J test	ELISA
Diagnostic accuracy, % (90% CI)	94.4% (90.8–97.0%)	94.4% (90.8–97.0%)
Sensitivity, % (90% CI)	94.4% (88.7–97.8%)	88.9% (81.9–93.8%)
Specificity, % (90% CI)	94.4% (88.7–97.8%)	100% (96.0–100%)

90% CI, 90% confidence interval; l‐HP J, l‐type Wako *Helicobacter pylori* antibody J.

### Comparison of concordance rates with ELISA (conventional test)

The number of subjects who were positive for anti‐*H. pylori* antibody using the ELISA and l‐HP J test were 80 and 90, respectively. The concordance rates between l‐HP J test and ELISA were high (*κ* = 0.8444) (Table 3). Subsequently, using a McNemar's test, we analyzed the tendency for positive or negative results for *H. pylori* between the l‐HP J test and ELISA. The results revealed that ELISA significantly tended to evaluate the test cases as negative (*P* = 0.0129) (Table 3).

**Table 3:** Investigation of concordance rates between the l‐type Wako *Helicobacter pylori* antibody J test and the ELISA

	ELISA positive	ELISA negative	Total	*P* value	*κ*
l‐HP J test positive	78	12	90	0.0129	0.844 (0.767–0.922)
l‐HP J test negative	2	88	90		
Total	80	100	180		

McNemar's test.

l‐HP J, l‐type Wako *Helicobacter pylori* antibody J.

### Investigation of cases misdiagnosed by the l‐HP J test

Cases misdiagnosed by the l‐HP J test included five false‐negative cases (5.6%) and five false‐positive cases (5.6%) (Table 4). Among the false‐positive cases, one subject had a high titer, while the other four subjects had titers slightly above the cutoff value. All false‐negative subjects had open‐type mucosal atrophy.

**Table 4:** Misdiagnosed cases by the l‐type Wako *Helicobacter pylori* antibody J test

			*H. pylori* antibody measurement result				Urea breath test		Endoscopic findings
Case number	Sex	Age	l‐HP J (U/mL)	Judgment	ELISA (U/mL)	Judgment	Change rate (‰)	Judgment	Kimura‐Takemoto classification
False‐positive case of l‐HP J									
1	M	68	4.1	+	3.3	−	0.5	−	No atrophy
2	F	63	4.3	+	3	−	0.6	−	No atrophy
3	M	72	5.2	+	3	−	0.5	−	No atrophy
4	F	54	5.2	+	5.5	−	0.0	−	No atrophy
5	M	73	13.6	+	4.7	−	0.2	−	No atrophy
False‐negative case of l‐HP J									
6	F	63	3.9	−	14	+	3.3	+	O‐3
7	F	76	3.1	−	15.5	+	6	+	O‐3
8	F	69	3.5	−	4.8	−	9.5	+	O‐3
9	M	84	2.7	−	7.5	−	59.2	+	O‐1
10	F	67	3.24	−	3	−	2.7	+	O‐2
False‐negative case of ELISA									
1	M	28	16.56	+	6.4	−	5.7	+	O‐1
2	M	57	7.2	+	9.7	−	13.9	+	C‐2
3	F	69	3.5	−	4.8	−	9.5	+	O‐3
4	M	70	5.9	+	5.9	−	3.9	+	O‐1
5	F	39	16.1	+	6.9	−	5	+	C‐1
6	M	84	2.7	−	7.5	−	59.2	+	O‐1
7	F	67	3.24	−	3	−	2.7	+	O‐2
8	M	56	18.2	+	3	−	6.8	+	O‐1
9	M	66	5.5	+	5.5	−	5.1	+	O‐1
10	M	70	18.6	+	8.4	−	24.1	+	O‐2

l‐HP J, l‐type Wako *Helicobacter pylori* antibody J.

## Discussion

Invasive diagnostic methods for *H. pylori* infection listed in the Japanese Society for Helicobacter Research guidelines^6^ include the rapid urease test, microscopic test, and culture test; and noninvasive methods include UBT, serous *H. pylori* antibody test, and feces *H. pylori* antigen test. Among them, the serous *H. pylori* antibody test is relatively convenient and widely used. A prevailing method for measuring this antibody is ELISA.

In this study, we investigated a newly launched kit that can be used with a general‐purpose automatic analyzer: the l‐HP J test kit. The l‐HP J test can be performed using the commercially available automated analyzers, such as Hitachi 7180 (Hitachi High‐Tech), JCA‐BM6050 (JEOL Ltd., Tokyo, Japan), ARCHITECT c16000 (Canon Medical Systems, Tokyo, Japan), and DxC700AU (Beckman Coulter K.K., Tokyo, Japan). The l‐HP J test was launched in April 2016 by FUJIFILM Wako Pure Chemical Corporation and is based on the latex agglutination turbidimetric immunoassay. To our knowledge, this is the first report about the diagnostic validity of this newly launched kit. We demonstrated that the diagnostic accuracy, sensitivity, and specificity of the l‐HP J test were approximately 95%, and the concordance rate between the l‐HP J test and conventional ELISA was high with a κ statistic of 0.8444. AUC of the two kits showed high diagnostic capability and showed no significant differences.

Although ELISA showed high specificity, the lower limits of 90% CIs for sensitivity of ELISA did not exceed the threshold value of 85%. In addition to this, ELISA significantly tended to evaluate the test cases as negative. From these findings, it can be considered that ELISA test has a higher rate of false‐negative results, and l‐HP J test is a balanced kit.

The latex agglutination turbidimetric immunoassay is superior to ELISA in terms of throughput and saving labor, and is expected to greatly contribute to rapid testing. Kita et al. demonstrated that the diagnosis of *H. pylori* infection is improved when two strains from genotypes commonly seen in the Japanese (#3 and #5)^8^ are used. The l‐HP J test we evaluated in this study used the same two antigens as the chemiluminescent test. Our findings showed that the l‐HP J test is no less accurate than ELISA, and is a balanced kit. About the false‐positive and false‐negative cases detected using the l‐HP J test, all but one false‐positive case were low‐positive titers (between 4 and 8.7 U/mL). It has been reported that approximately 10% of individuals with low‐positive titers detected using the l‐HP J test are uninfected.^9^ Thus, we believe that this problem arises due to the established cutoff value. About the false‐negative cases, there are many subjects with open‐type mucosal atrophy, suggesting that the antibody titers are lower in such cases. It is important to use additional tests, such as the UBT, to diagnose *H. pylori* infection correctly if the l‐HP J test is not consistent with the endoscopic findings, such as in the following two cases. First, when an individual has an open‐type mucosal atrophy and endoscopic findings suggest current infection, but the l‐HP J test result is negative; and second, when endoscopic findings suggest uninfection, but the l‐HP J test result is low‐positive titers (between 4 and 8.7 U/mL).

In the present study, we considered both, the results of the UBT that has a high diagnostic accuracy,^10^ and endoscopic findings based on the Kyoto classification of gastritis. We then diagnosed the presence or absence of *H. pylori* infection in those subjects whose UBT results and endoscopic findings were consistent. Therefore, subjects who were previously infected with *H. pylori* and those who had type A gastritis could be excluded from the study. Moreover, this was a prospective study and antibody measurements by both kits were performed in‐house, which increase the reliability of the study.

There are some limitations of the study. First, in some subjects, fresh plasma was used for detection using ELISA and part of the l‐HP J test, while stored serum (within 4 weeks) was used for the l‐HP J test; therefore, specimens with different conditions were used for these subjects. However, supplemental data from FUJIFILM Wako Pure Chemical Corporation showed no difference in the titers between fresh plasma and stored serum (within 4 weeks) under conditions of −20°C. Second, we used the UBT as the gold standard for diagnosing *H pylori* infection; however, its accuracy is not 100%.^10^ Third, we investigated only noninfected and currently infected cases, not previously infected cases. Therefore, a further study should be conducted to analyze the association between previously infected cases and antibody titer of the l‐HP J test. Fourth, the evaluation of *H. pylori* status was performed based on UBT result and endoscopic findings, not histological result. However, endoscopic findings used in this study reportedly have high diagnostic odds ratio and are helpful in the diagnosis of current *H. pylori* infection.^6^, ^7^ Therefore, we believe that the evaluation of *H. pylori* status in this study is reliable.

In conclusion, the l‐HP J test has a high diagnosis performance, and is not inferior to ELISA. The l‐HP J test is useful, especially when screening for *H. pylori* infection, due to its high sensitivity.
